# Supplementary figures and images for: Senecavirus A-induced glycolysis facilitates virus replication by promoting lactate production that attenuates the interaction between MAVS and RIG-I
Source: PLoS Pathog. 2023 May 1;19(5):e1011371. doi: 10.1371/journal.ppat.1011371 (PMC10174517; doi:10.1371/journal.ppat.1011371)

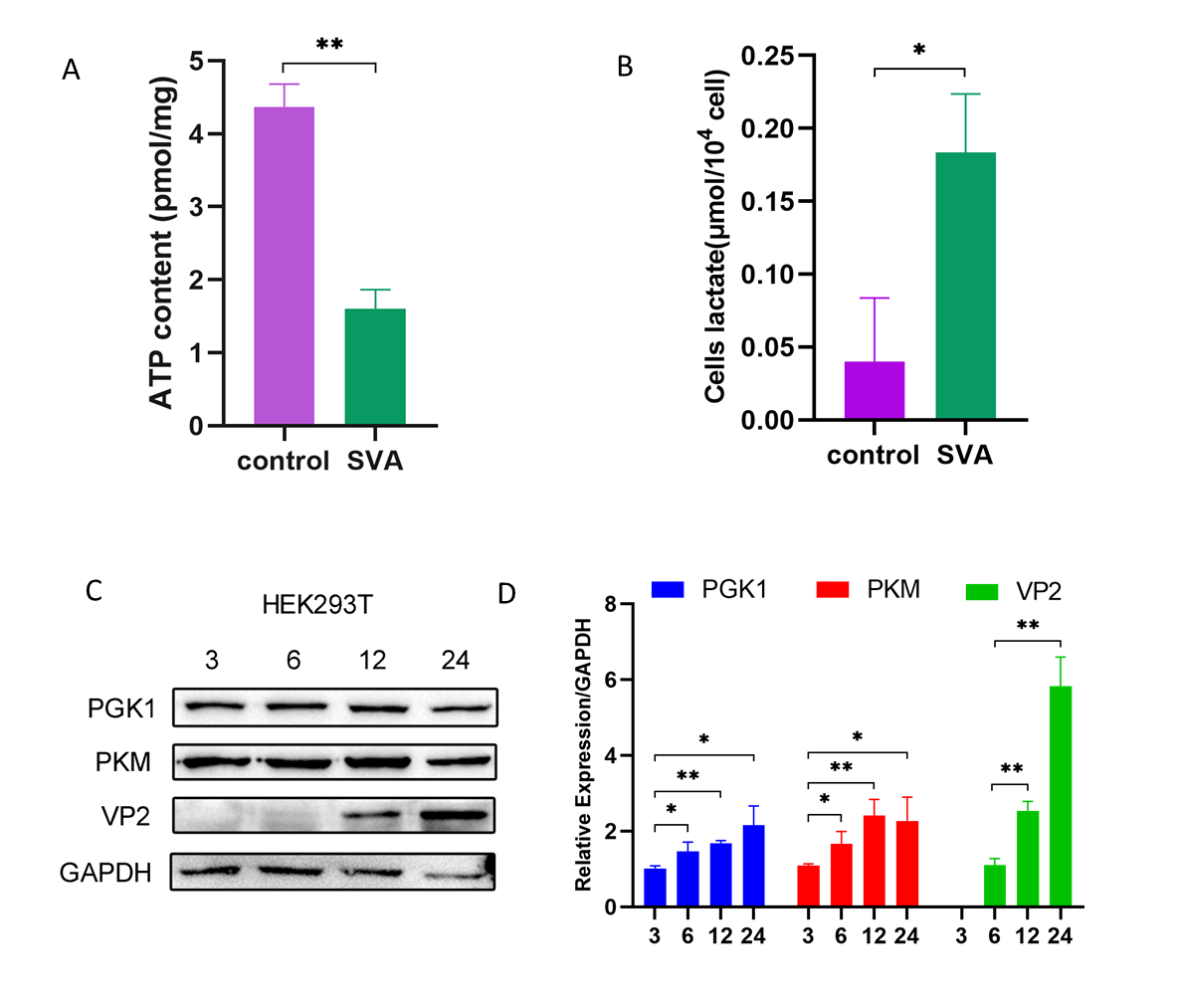

Supplement: S1 Fig — (A, B) HEK293T cells were infected with SVA at an MOI of 1. Cell lysates were collected at 48 h.p.i. and intracellular ATP levels and the lactate were measured. (C) Cells were infected with SVA at an MOI of 1 and lysed in RIPA buffer at 48 h.p.i. The expression levels of PGK1, PKM, and VP2 were analyzed by western blot. (D) The grayscale analysis of PGK1, PKM, and VP2 protein. All data represent the means ± SD (Student’s t test) (*P < 0.05, **P < 0.01, ns, not significant). (TIF) [file ppat.1011371.s001.tif]

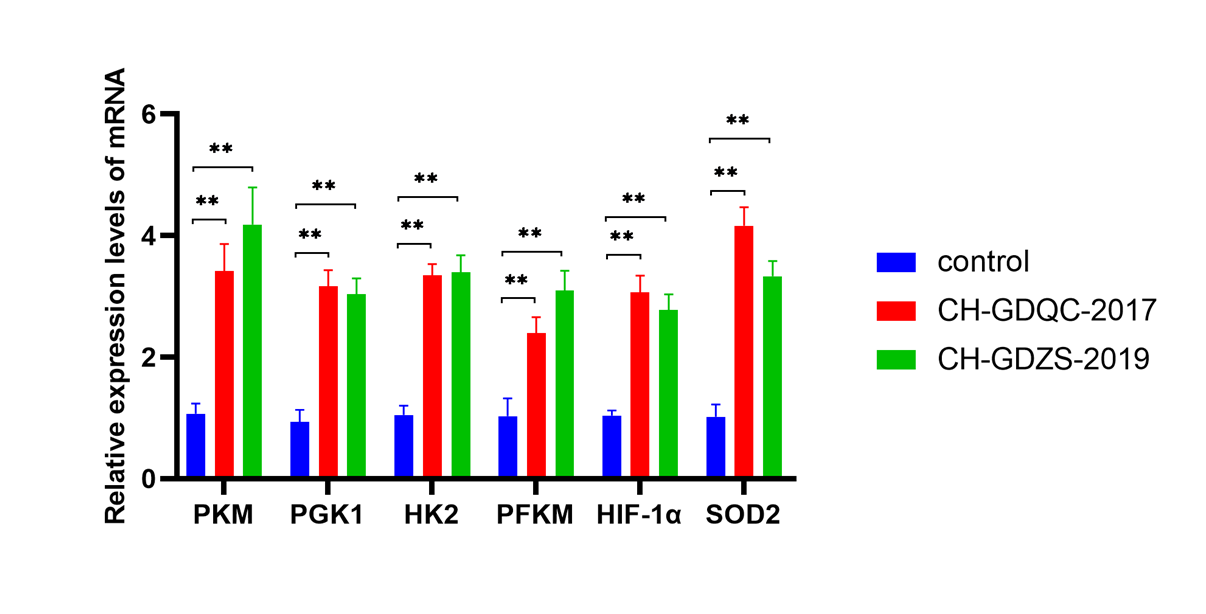

Supplement: S2 Fig — (TIF) [file ppat.1011371.s002.tif]
