# Supplementary material for: Senecavirus A-induced glycolysis facilitates virus replication by promoting lactate production that attenuates the interaction between MAVS and RIG-I
Source: PLoS Pathog. 2023 May 1;19(5):e1011371. doi: 10.1371/journal.ppat.1011371 (PMC10174517; doi:10.1371/journal.ppat.1011371)
Supplement: S1 Table — (DOC) [file ppat.1011371.s004.doc]

**S1 Table: Primer sequences for qRT-PCR**

| **primers** | **Sequence (5’-3’)** |
| --- | --- |
| Swine-SOD2-F | GGCCTACGTGAACAACCTGA |
| Swine-SOD2-R | TGATTGATGTGGCCTCCACC |
| Swine-PKM-F | CACCTCACACCAGTTCCTCC |
| Swine-PKM-R | GATGGGGCTCAGGTAGATGC |
| Swine-PFKM-F | GGGCCGATGCTGCCTATATT |
| Swine-PFKM-R | TGTCGAAGATGCCTTTCCCC |
| Swine-HK2-F | GCTCAACCATGACCAAGTGC |
| Swine-HK2-R | GTGGTAGCTCCAAGCCCTTT |
| Swine-PGK1-F | GCTGGACGTGAAGGGAAAGA |
| Swine-PGK1-R | CTGACTTGGCTCCGTTGTCT |
| Swine- HIF-1α-F | CAGCCAGATGATCGTGCAAC |
| Swine- HIF-1α-R | CCATTGATTGCCCCAGGAGT |
| Swine-β-actin-F | TCTGGCACCACACCTTCT |
| Swine-β-actin-R | TGATCTGGGTCATCTTCTCAC |
| Swine-IFNβ-F | TGATGGGCAGATGGATGACC |
| Swine-IFNβ-R | AGGCACAGCTTCTGTACTCC |
| Swine-RIG-I-F | AACTCCCAGTGTATGAGCAGC |
| Swine-RIG-I-R | GGAATTGTCCCATTGGTAAGA |
| Swine-IFNα-F | ATCTGCTCTCTGGGCTGTGA |
| Swine-IFNα-R | CATGGCTTGAGCCTTCTGGA |
| Swine-ISG15-F | CAGAGACCCACTGAGCATCC |
| Swine-ISG15-R | GCGTCAGCCAGACCTCATAG |
| Swine-IL-6-F | GCTGCTTCTGGTGATGGCTA |
| Swine-IL-6-R | TGAGGTGGCATCACCTTTGG |
| Swine -IFIT1-F | GGTCTTGGAGGAGATTGAG |
| Swine -IFIT1-R | TAACCAGCCTTCTCACCTC |
| SVA-VP2-F | TCAACCCACCACCACTTTTA |
| SVA-VP2-R | CTGCCACCCGCACTTCATTA |
| Mice-IFNβ-F | TCCGAGCAGAGATCTTCAGGAA |
| Mice-IFNβ-R | TGCAACCACCACTCATTCTGAG |
| Mice-IL6-F | AGTTGCCTTCTTGGGACTGATG |
| Mice-IL6-R | GGGAGTGGTATCCTCTGTGAAGTCT |
| Mice-IFNα-F | CCTGAACATCTTCACATCAAAGGA |
| Mice-IFNα-R | AGCTGCTGGTGGAGGTCATT |
| Mice-β-actin-F | TATCGCTGCGCTGGTCGT |
| Mice-β-actin-R | CCACGATGGAGGGGAATACAG |
| Mice-RIG-I-F | GTGGACTTTGTGAAGCCATCG |
| Mice-RIG-I-R | GCACCTGCCATTCTCCCTTTA |
| Mice-ISG15-F | TGGTACAGAACTGCAGCGAG |
| Mice-ISG15-R | AGCCAGAACTGGTCTTCGTG |
