# Supplementary material for: Senecavirus A-induced glycolysis facilitates virus replication by promoting lactate production that attenuates the interaction between MAVS and RIG-I
Source: PLoS Pathog. 2023 May 1;19(5):e1011371. doi: 10.1371/journal.ppat.1011371 (PMC10174517; doi:10.1371/journal.ppat.1011371)
Supplement: Attachment — Submitted filename: Point to point response.doc [file ppat.1011371.s005.doc]

**PLoS Pathogens Manuscript # PPATHOGENS-D-23-00018**

**Point-to-point Response**

“Senecavirus A-induced glycolysis facilitates virus replication by promoting lactate production that attenuates the interaction between MAVS and RIG-I” by Huizi Li, Cunhao Lin, Wenbao Qi, ZhenZhen Sun, Zhenxin Xie, Weixin Jia, Zhangyong Ning

Many thanks for insightful and constructive comments from editors and reviewers. We are pleased that reviewers found that the manuscript *is interesting*. Reviewers also felt that the paper adds “*a new idea for controlling the disease from the aspect of metabolic control*”. The manuscript was carefully revised in accordance with comments from editors and reviewers and the revision guidelines. We have experimentally addressed the points and have revised the manuscript by adding new figure panels as well adding related text content. Here below is our point-to-point response on revision according to comments.

**Editor Concerns :**

1. We encourage you to corroborate the main cell culture findings (Fig 5D-F) with TCDID_50_ or plaque assay. From the RNA and protein levels the reader can clearly see the trends but not the effect size. To which extent was the replication inhibited?

**Reply:** Many thanks. We have corroborated the main cell culture findings with TCDID_50_ by the New Figure 5F in our revised manuscript to better show the effect size of lactate and oxamate on SVA replication.

2. Does glycolysis facilitate senecavirus reproduction solely via RIG-I signaling or other pathways involved as well? To this end, an experiment with infection in RIG-I knockdown (e.g., shRNA or CRISPRi) plus/minus glycolysis inhibitors plus/minus lactate would be very informative.

**Reply:** Many thanks. RIG-I knockout (RIG-I-KO) PK-15 cells were prepared using CRISPR-Cas9 systems to detect the effects of lactate and oxamate on IFNs in our revised manuscript. The results of experiment with infection in RIG-I-KO PK-15 cells plus oxamate or lactate showed that glycolysis facilitate senecavirus reproduction mainly via RIG-I signaling (New Fig. 6). There was no significant difference in IFNβ, ISG15, IFNα, and IFIT1 between SVA infected oxamate-RIG-I-KO and RIG-I-KO groups (New Fig 6D-6G). Lactate has a slight inhibitory effect on ISG15, IFNα, and IFIT1 in RIG-I-KO PK-15 cells, however, there was no significant difference compared with the SVA infected RIG-I-KO cells (New Fig 6E-6G), indicating that the regulation of IFNs by lactate was mainly through RIG-I signaling.

The related contents of “Generation of RIG-I knockout PK-15 cells by CRISPR-Cas9-mediated genome editing” also have been added to materials and methods and Figure legend of New Figure 6 was also added the related content in our revised manuscript. Also, we added the contents “The knockout of RIG-I weakened the regulatory effect of oxamate and lactate on IFNs production and lactate slightly inhibit the production of IFNs in RIG-I-KO cells, suggesting that lactate may inhibit IFNs by other pathways, however, it mainly relies on RIG-I to manipulate SVA-induced innate immunity.” In discussion in our revised manuscript.

3. More information on tissue pathology in mice is required, as suggested by Reviewer #2.

**Reply:** Many thanks. The results of histopathological detection was added as the New Figure 8 in our revised manuscript. The results showed that mice treated by high glucose and lactate promote the damage of SVA infection with the degeneration and necrosis of mucosal epithelial cells and shedding of intestinal villi, vacuolar degeneration of renal tubular epithelial cells, and the decrease of lymphocytes in splenic corpuscle and white pulp. In addition, lactate increased the vacuolar degeneration and necrosis with fewer in quantity of liver cells. Histopathological changes were reduced in oxamate and oxamate + lactate group compared with the high glucose and lactate group. And, significant difference in pathological lesions was not observed in group infected by SVA.

The related contents of histopathological detection have been added to “animal experiments” was added in materials and methods and the Figure legend of New Figure 8 was also added in our revised manuscript. Also, we added the contents “The results of histopathology in animal experiment also support this” In discussion in our revised manuscript.

4. Lines 251-254: Please correct name superscripts of virus proteins, e.g. 2B, 2C etc, (see PMID: 28884666  for guidance and links).

**Reply:** Many thanks. We had change “2B^pro^, 2C^pro^, 3C^pro^, 3D^pro^” to “2B proteinase (2B^pro^), 2C proteinase (2C^pro^), 3C proteinase (3C^pro^), 3D proteinase (3D^pro^)”.

**Comments from reviewer #1:**

1. Its clinical value to control SVA in pigs remains further confirmed. Are the findings of this study limited to the single strain used or to all SVA strains? Is there any unique changes in the CH-GDFS-2018 strain? This needs to be discussed.

**Reply:** Many thanks. Supplemengtary Figure S2 (S2 Fig) was provided to show the findings of the virus was not limited to the single strain. And we discussed this in discussion part of our revised manuscript. The added contents was “CH-GDFS-2018 is a wild type virus isolated by our group in 2018 with recombinant features [39], in order to verify whether the clinically isolated strains have the same characteristics that promote glycolysis, the mRNA levels of HIF1α, PGK1, HK2, SOD2, PKM and PFKM after cell infected with CH-GDQC-2017 [40] and CH-GDZS-2019 [41] were verified, and the results showed that these strains had similar abilities to cause glycolysis (S2 Fig), indicating that glycolysis induced by SVA infection is not unique to a particular strain.” . Also, the related references 39,40 and 41 was also added to the references in our reviosed manuscript. The added reference are as follows:

**39. Liu J, Zha Y, Li H, Sun Y, Wang F, Lu R, et al. Novel Recombinant Seneca Valley Virus Isolated from Slaughtered Pigs in Guangdong Province. Virol Sin. 2019;34(6):722-724. pmid: 31240619.**

**40. Liu J, Ren X, Li Z, Xu G, Lu R, Zhang K, et al. Genetic and phylogenetic analysis of reemerged novel Seneca Valley virus strains in Guangdong province, 2017. Transbound Emerg Dis. 2018;65(3):614-617. pmid: 29461010.**

**41.	Liu J, Guo Q, Li H, Yu X, Liu B, Zhao B, et al. Genomic diversity and recombination of Seneca Valley viruses emerged in pig herds in Guangdong Province during 2019. Virus Genes. 2020;56(5):642-645. pmid: 32447588.**

2. Minor: Page 2, abbreviations of many molecules and pathways needed spell out. The manuscript needs to be improved since several places lack of scientific writing. Lines 241-243, what is the meaning of “this first outbreak”?

**Reply:** We appreciate the reviewer’s comments. As suggested, we have spell out the abbreviations of molecules and pathways, and corrected “this first outbreak” to “the first outbreak”.

**Comments from reviewer #2:**

1. Cell lines commonly used for SVA include PK-15 and HEK293T cells. Whether SVA infection-mediated glycolysis exists in non-porcine origin cells, HEK293T? It is better to clearly state.

**Reply:** Thanks for the reviewer’s comments. SVA infection-mediated glycolysis also exists in HEK293T cells. Supplemengtary Figure S1 (S1 Fig) was provied to show this in our revised manuscript. Also, we added this results in our revised manuscript.

2. In Figure 8, schematic overview, this figure has clearly defined the whole research context. It is recommended to remove the dotted line outside the cell membrane.

**Reply:** According to the reviewer’s suggestion, we have removed the dotted line outside the cell membrane in Figure 8, and rearranged this figure as Fig 9.

3. In animal experiments, the results showed the difference of virus load in tissues collected with various treatment in different groups. Readers would like to know whether this change of viral load caused by different treatment lead to pathological or histopathological change? This is related to the effect of different factors on the pathogenicity of the virus.

**Reply:** Many thanks. The detailed information can be seen in the third reply to the concerns of the editor. The results of histopathological detection was added as the New Figure 8 in our revised manuscript.

Minor: 1. Abstract Page 2, line 35: Please specify what experiments while not “Further”; 2. Page 7, lines: 141-145. Please note that contents in brackets for 2DG, oxamate and DCA was information belongs to the methods;

3. Page 25, lines: 605. “proteins” should be “detected proteins”.

**Reply:** Many thanks. We have revised these. Detailed revision see our revised manuscript (Tracked manuscript).

**Comments from reviewer** #3:

1. It is mentioned that glycolysis promotes viral replication, but for viral replication, the authors measures the expression of SVA VP2 protein. It suggests that viral titers should be preferably determined. Otherwise, explain the feasibility of VP2 protein test.

**Reply:** Thanks for the reviewer’s comments. This question is similar in nature to the first concern of the editor. The detailed information can be seen in the first reply to the concerns of the editor. The results of viral titers was provied in our revised manuscript. In addition, VP2 protein, as one of the structural proteins of SVA, is necessary for virus replication and assembly into complete virions. VP2 can be used to detect the replication of SVA. The detailed information included in the following reference.

**Chen Y, Zhong W, Xie Z, Li B, Li H, Gao K, Ning Z. Suppressor of cytokine signaling 1 (SOCS1) inhibits antiviral responses to facilitate Senecavirus A infection by regulating the NF-κB signaling pathway. Virus Res. 2022, 313:198748.**

2. In Figure 1, differences in mRNA levels of some proteins related to glycolysis such as HK2, PFKM, PKM, PGK1 and HIF-1α were detected after SVA virus infection, but only PGK1 and PKM were detected during protein level detection. Please explain why the latter two were selected for protein level detection.

**Reply:** Thanks for the reviewer’s comments. We refer to the study of "Influenza A Virus (H1N1) Infection Induces Glycolysis to Facilitate Viral Replication" to detect the proteins in the glycolysis process. While, at present, there are only commercial human HK2, PKM and PDK3 antibodies and the protein homology of human and swine HK2 is only 24.1%, we cannot detect the HK2 protein of PK-15 cells using human antibody. Although the protein homology of PDK3 between human and swine was high, but it is difficult to detect in PK-15 cells. In view of the limitations of antibodies, we detected PGK1 and PKM proteins in glycolytic enzymes.

3. Minor: FIG. 1C, the ordinate icon "Relative viral RNA" should be modified to "Relative expression levels of mRNA".

**Reply:** Thanks for the reviewer’s comments. We have revised this in our revised manuscript.
