# Supplementary material for: Senecavirus A-induced glycolysis facilitates virus replication by promoting lactate production that attenuates the interaction between MAVS and RIG-I
Source: PLoS Pathog. 2023 May 1;19(5):e1011371. doi: 10.1371/journal.ppat.1011371 (PMC10174517; doi:10.1371/journal.ppat.1011371)
Supplement: Attachment — Submitted filename: Point to point response.doc [file ppat.1011371.s006.doc]

**PLoS Pathogens Manuscript # PPATHOGENS-D-23-00018**

**Point-to-point Response**

“Senecavirus A-induced glycolysis facilitates virus replication by promoting lactate production that attenuates the interaction between MAVS and RIG-I” by Huizi Li, Cunhao Lin, Wenbao Qi, ZhenZhen Sun, Zhenxin Xie, Weixin Jia, Zhangyong Ning

Many thanks for comments from editors and reviewers. We are pleased that “the reviewers appreciated the attention to an important topic”. We have made revisions based on comments from editors and reviewers, here below is our point-to-point response on revision according to comments.

**Editor Concerns:**

Please fix the protein names that are misrepresented: Lines 251/254: “2B proteinase (2Bpro), 2C proteinase (2Cpro), 3C proteinase (3Cpro), 3D proteinase (3Dpro)”.

**Reply:** Many thanks. The protein names of “2B^pro^, 2C^pro^, 3C^pro^, 3D^pro^” have changed to “2B, 2C^hel^, 3C^pro^, 3D^pol^” in our revised manuscript.

**Reviewer #3:**

In Figure 3 E and G. For sonsistency, it is suggested to place HG column on the left and LG column on the right.

**Reply:** Many thanks. As suggested, HG column of FIG3 G was placed on the left, and LG column on the right in our NEW Fig 3 for consistency.
